# Anterograde interference emerges along a gradient as a function of task similarity: A behavioural study

**DOI:** 10.1111/ejn.15561

**Published:** 2021-12-20

**Authors:** Raphaël Hamel, Jean‐François Lepage, Pierre‐Michel Bernier

**Affiliations:** ^1^ Département de kinanthropologie, Faculté des sciences de l'activité physique Université de Sherbrooke Sherbrooke Québec Canada; ^2^ Département de pédiatrie, Faculté de médecine et des sciences de la santé Université de Sherbrooke; Centre de Recherche du Centre Hospitalier Universitaire de Sherbrooke Sherbrooke Québec Canada

**Keywords:** anterograde interference, competing memories, implicit adaptation, motor memory, retention

## Abstract

Anterograde interference emerges when two opposite (B → A) or identical tasks (A → A) are learned in close temporal succession, suggesting that interference cannot be fully accounted for by competing memories. Informed by neurobiological evidence, this work tested the hypothesis that interference depends upon the degree of overlap between the neural networks involved in the learning of two tasks. In a fully within‐subject and counterbalanced design, participants (*n* = 24) took part in two learning sessions where the putative overlap between learning‐specific neural networks was behaviourally manipulated across four conditions by modifying reach direction and the effector used during gradual visuomotor adaptation. The results showed that anterograde interference emerged regardless of memory competition—that is, to a similar extent in the B → A and A → A conditions—and along a gradient as a function of the tasks' similarity. Specifically, learning under similar reaching conditions generated more anterograde interference than learning under dissimilar reaching conditions, suggesting that putatively overlapping neural networks are required to generate interference. Overall, these results indicate that competing memories are not the sole contributor to anterograde interference and suggest that overlapping neural networks between two learning sessions are required to trigger interference. One discussed possibility is that initial learning modifies the properties of its neural networks to constrain further plasticity induction and learning capabilities, therefore causing anterograde interference in a network‐dependent manner. One implication is that learning‐specific neural networks must be maximally dissociated to minimize the interfering influences of previous learning on subsequent learning.

## Graphical abstract

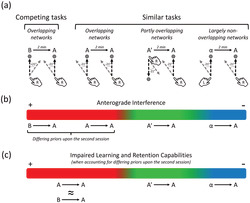

**Abbreviations**
ATP: adenosine triphosphate
FF: force field
MAD: median absolute deviation
MT: movement time
NMDA: *N*‐methyl‐d‐aspartate
PV: peak velocity
RM ANOVA: repeated measures analysis of variance
RT: reaction time
SYH: synaptic homeostasis hypothesis

## 1 INTRODUCTION

Anterograde interference refers to the phenomenon whereby initial learning of task A interferes with the subsequent learning and retention of an opposing task B (Herszage & Censor, 2018; Lerner et al., 2020; Robertson, 2018). The most common interpretation for this phenomenon is that anterograde interference emerges because the memories of A and B compete for limited biological resources required for their encoding in functionally overlapping neural networks (Cantarero, Lloyd, & Celnik, 2013; Cantarero, Tang, et al., 2013; Herszage & Censor, 2018; Robertson, 2018). Although the notion that the memories of A and B share functionally overlapping networks appears consensual (Herszage & Censor, 2018; Robertson, 2018), recent work suggests that interference may not merely arise because of a competition between the two memories (Hamel et al., 2021).

In a series of experiments, Hamel et al. (2021) had participants adapt to the same gradually introduced visual deviation twice over two distinct sessions (A → A) and measured retention levels through immediate reach aftereffect assessment. The results revealed that initial learning of A reliably interfered in a time‐, dose‐ and learning‐dependent manner by preventing the emergence of savings and by interfering with retention levels upon the second session as compared with the first one, indicative of the presence of anterograde interference. Specifically, the results of a first experiment revealed that retention impairments were only observable when a 2‐min, but not 1 h or 24 h, interval separated the two learning sessions, indicating that such impairments only occur during a short time window. The results of a second experiment indicated that adding a third learning session amplified the retention impairments while those of a third experiment revealed that the impairments are learning specific and independent of the accumulation of fatigue. Globally, these results suggest that anterograde interference—as assessed through a lack of savings and retention impairments—can emerge even when there is no competition per se between identical tasks learned in close temporal proximity.

Based on extensive neurobiological evidence (Keck, Hübener, et al., 2017; Kukushkin & Carew, 2017; Lee & Kirkwood, 2019; Smolen et al., 2016), one interpretation of the results from Hamel et al. (2021) is that interference arises because the same neural networks—or largely overlapping ones—are recruited twice by the two identical learning sessions. Specifically, through the induction of synaptic plasticity, learning is known to transiently perturb the metabolic homeostasis of its associated neural network (Keck, Hübener, et al., 2017; Kukushkin & Carew, 2017; Lee & Kirkwood, 2019; Smolen et al., 2016). To prevent further deviations from homeostasis, biological constraints quickly emerge to limit further synaptic plasticity induction in the same network (Keck, Hübener, et al., 2017; Kukushkin & Carew, 2017; Lee & Kirkwood, 2019; Smolen et al., 2016). In this light, one possibility is that anterograde interference emerges when a first learning session perturbs the homeostasis of a network that sufficiently overlaps with the one involved in a second learning session. Relevant to support this possibility is the sliding threshold model, an influential computational model of homeostatic plasticity which posits that the capacity for a neural network to induce synaptic plasticity is determined by its past neural activity (Keck, Hübener, et al., 2017; Lee & Kirkwood, 2019); the higher the past activity in a network, the more difficult it becomes for this same network to potentiate synaptic connections (Keck, Hübener, et al., 2017; Lee & Kirkwood, 2019) and, thus, to acquire and store new memories (Cirelli, 2017; Tononi & Cirelli, 2020). Directly supported by recent animal work (see Crossley et al., 2019), an important ramification of this model is that anterograde interference should scale with the degree of overlap between the neural networks involved in two learning sessions. Based on all the above evidence, this work tested the hypothesis that learning similar tasks, by recruiting overlapping neural networks, generates more anterograde interference than learning dissimilar ones. Importantly, in the context of this work, tasks are considered more similar when they require similar reaching movement directions and recruit similar effectors (see below).

**FIGURE 1:**
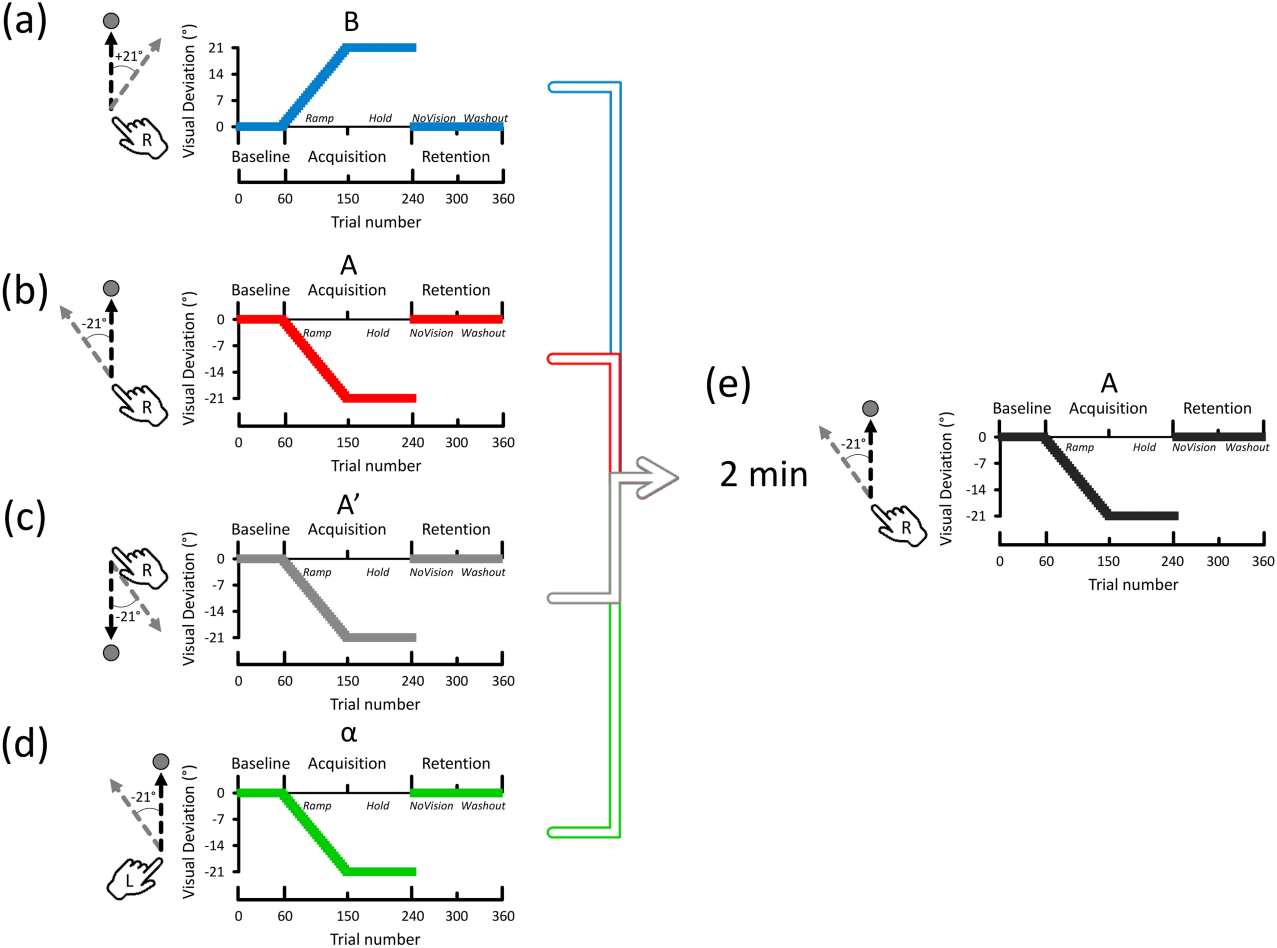
Conditions and procedures. The first session was manipulated so that the extent of putative overlap between the neural networks involved in the two sessions would differ. The second session (see panel (e)) was identical across conditions (right arm; upper targets; −21° visual deviation). (a) B → A condition: Right‐handed reaching movements towards upper targets while compensating for a +21° visual deviation (B) in the first session (Lerner et al., 2020). (b) A → A condition: Right‐handed reaching movements towards upper targets while compensating for a −21° visual deviation in both the first (A) and second sessions (A; Hamel et al., 2021). (c) A′ → A condition: Right‐handed reaching movements towards targets in the lower quadrant were performed while compensating for a −21° visual deviation in the first session (A′). (d) α → A condition: Left‐handed reaching movements towards upper targets were performed while compensating for a −21° visual deviation in the first session. (e) Second session: The second session was identical to the one depicted under panel (b) and was used as a test condition to evaluate the effects of the first session on subsequent learning and retention capabilities. The average of each of the three phases (Baseline [60 trials]; Acquisition [average of both Ramp and Hold; 180 trials]; Retention [average of both NoVision and Washout; 120 trials]) was calculated for the statistical analyses

In a fully within‐subject design meant to optimize statistical power (Algermissen & Mehler, 2018), right‐handed healthy participants (*n* = 24) adapted twice to the same task that consisted of a gradually introduced ±21° visual deviation over two implicit learning sessions occurring in close temporal proximity (2 min; Hamel et al., 2021). Importantly, conditions were fully counterbalanced across participants to control for order effects (Brooks, 2012). Also, each participant performed extensive familiarization sessions (100 trials) with both effectors before each experimental session for performance to revert to baseline levels and thus minimize potential between‐condition carryover effects (Brooks, 2012). Retention, measured through reaching aftereffects, was evaluated immediately following adaptation in each learning session. Evidence of performance impairment upon the second learning session as compared with the first one would be assumed to be reflective of the emergence of anterograde interference, which has been previously observed as impaired relearning (Lerner et al., 2020) and/or retention capabilities (Hamel et al., 2021). This is also similar to the evaluation of retrograde interference (i.e., B → A → B), which can also be measured as impaired relearning (Brashers‐Krug et al., 1996; Krakauer, 2005; Wigmore et al., 2002) and/or retention capabilities (Keisler & Shadmehr, 2010; Pekny et al., 2011; Yan et al., 2020). To manipulate the extent of overlap between task‐specific neural networks, four behavioural conditions were carried out (see Figure 1).

In the B → A condition (Figure 1a), participants learned opposite tasks by adapting to *opposite* visual deviations with their right upper limb to generate anterograde interference (Hinder et al., 2007; Lerner et al., 2020). Given that the neural networks involved in B and A largely overlap (Herszage & Censor, 2018; Robertson, 2018), substantial anterograde interference was expected to emerge from this condition. In the A → A condition (Figure 1b), participants learned identical tasks by adapting to the *same* visual deviation twice with their right upper limb, which would also recruit largely overlapping neural networks and thus induce meaningful levels of anterograde interference (Figure 1b). Here, the B → A and A → A conditions were compared together to ascertain that memory competition is not mandatory for anterograde interference to emerge (Hamel et al., 2021). Given that both the B → A and A → A conditions would recruit largely overlapping neural networks, it was hypothesized that both the B → A and A → A conditions would generate similar levels of interference.

In the A′ → A condition (Figure 1c), participants learned similar tasks by adapting to the same visual deviation twice with their right upper limb but performed reaching movements in opposite directions from the first session (lower workspace quadrants) to the second one (upper workspace quadrants). The manipulation of reaching direction is known to involve different directionally tuned neuronal populations within cortical regions of the same hemisphere (Cowper‐Smith et al., 2010; Eisenberg et al., 2010; Fabbri et al., 2010; Haar et al., 2015; Mahan & Georgopoulos, 2013; Tanaka et al., 2018; Toxopeus et al., 2011), thus leading to partial segregation of the neural networks involved in the two tasks. In support, converging lines of evidence indicate that motor adaptation in one workspace direction poorly generalizes to other workspace directions (Krakauer et al., 2000; Rezazadeh & Berniker, 2019; Schween et al., 2018). As a result, the A′ → A condition was conceptualized as yielding an intermediate level of overlap between the neural networks.

Finally, in the α → A condition (Figure 1d), participants learned similar tasks by adapting to the same visual deviation twice but performed the first and second learning sessions with their left and right arms, respectively. The use of different limbs is associated with the recruitment of cortical regions within opposite hemispheres (Bernier et al., 2012; Chang et al., 2008; Gallivan et al., 2011; Levy, 1969; Serrien et al., 2006; Welniarz et al., 2015; Yttri et al., 2014) and thus leads to a relatively large degree of segregation between the neural networks involved in the two tasks. In support, between‐limb transfer of motor adaptation is known to be minimal when the sensorimotor perturbation is gradually introduced (Malfait & Ostry, 2004; Werner et al., 2019). As a result, the α → A condition was thus conceptualized as yielding the lowest level of overlap between the neural networks.

Altogether, the above neurophysiological and behavioural evidence indicates that neural networks would overlap to a large extent in both the B → A and A → A conditions, moderate extent in the A′ → A condition and minimal extent in the α → A condition. Given that anterograde interference is expected to emerge as a function of the overlap in learning‐specific neural networks, it was hypothesized that the A → A, A′ → A and α → A conditions would, *respectively*, generate meaningful (Cohen's *d*_z_ of ~.8), modest (Cohen's *d*_z_ of ~.5) and negligible (Cohen's *d*_z_ of ~.3) amounts of anterograde interference. Finally, it is worthy to note that each session occurred at the same time of day to control for circadian influences on learning capabilities (Frank, 2016; Lehr et al., 2021) and at least 24 h separated each session.

## 2 RESULTS

**FIGURE 2:**
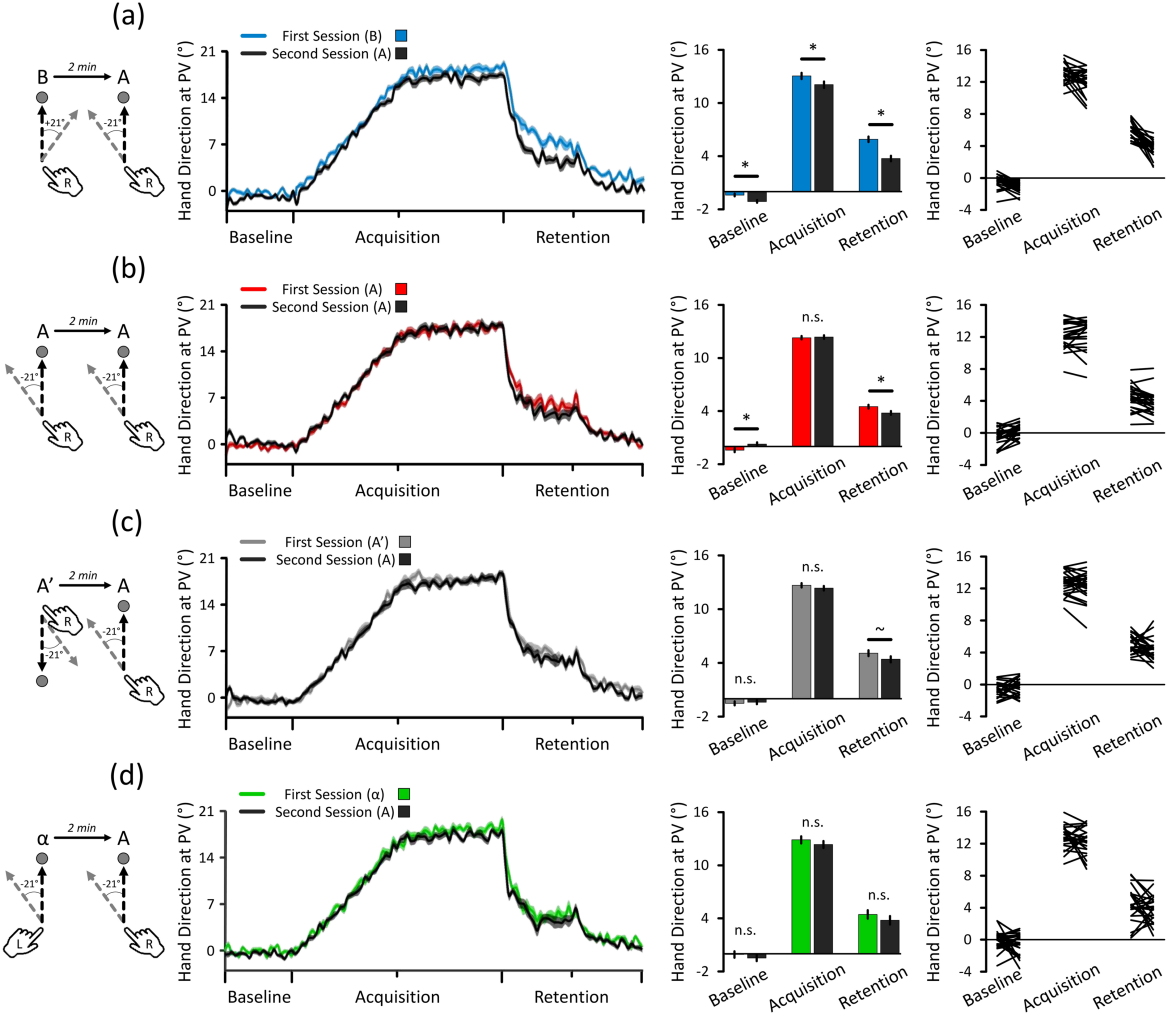
Hand Direction at peak velocity (PV) for all conditions. The leftmost column depicts the procedures of each condition while the rightmost column represents the individual data contained in the adjacent bar graphs. (a) B → A condition. Note that the data of Acquisition and Retention represent the absolute values of Hand Direction at PV (not for Baseline). The results revealed that initial learning of B induced negative reach biases at Baseline (*d*_z_ = .969 [.475 1.450]) and interfered with subsequent Acquisition (*d*_z_ = .559 [.127 .985]) and Retention of A (*d*_z_ = 1.392 [.818 1.949]). (b) A → A condition. The results revealed that initial learning of A induced a positive reach bias at Baseline (*d*_z_ = .622 [.178 1.054]), did not enhance Acquisition (*d*_z_ = .079 [−.323 .478]), but interfered with the Retention of the subsequent learning of A (*d*_z_ = .681 [.230 1.121]). (c) A′ → A condition. The results revealed that initial learning of A′ neither induced a reach bias at Baseline (*d*_z_ = .113 [−.290 .513]) nor interfered with Acquisition (*d*_z_ = .260 [−.150 .664]) but modestly impaired the Retention of A (*d*_z_ = .410 [−.011 .823]). (d) α → A condition. The results revealed that initial learning of α did not induce a reach bias at Baseline (*d*_z_ = .217 [−.190 .620]) and did neither interfere with the Acquisition (*d*_z_ = .267 [−.143 .672]) nor Retention of the second session (*d*_z_ = .273 [−.138 .678]). Note that the presence of a gradient is quantitatively supported by the gradually decreasing Retention difference effect sizes across the four conditions. Asterisks (*) and tildes (~) represent significant and marginal differences, respectively. For all panels, the mean ± 95% within‐subject confidence intervals are depicted

To analyse the data, 4 Conditions (B → A, A → A, A′ → A, α → A) * 2 Sessions (First, Second) * 3 Phases (Baseline, Acquisition, Retention; see the caption of Figure 1) repeated measures ANOVAs (RM ANOVAs) were conducted separately on Hand Direction at peak velocity (PV), reaction time (RT), endpoint accuracy, movement time (MT) and hit rates data (see the Supporting Information for additional details). Concerning Hand Direction at PV, data were first corrected for condition‐specific reach biases (see the Supporting Information). Then, the absolute rather than the raw Acquisition and Retention data of the B task were used for statistical analyses to render the data tied to the adaptation of the +21° deviation comparable with all other conditions where adaptation to a −21° occurred (see Figure 2a).

To decompose three‐way interactions, separate Sessions * Phases RM ANOVAs were conducted on each condition. Benjamini–Hochberg corrected pairwise comparisons were conducted to decompose main effects or interactions. The effect size values (Cohen's *d*_z_) are followed by their 95% confidence intervals (reported within square brackets). As recommended (Greenwald et al., 1996; Ho et al., 2019), emphasis is placed on the effect sizes rather than on the *p* values to interpret the results. This allows to quantitatively evaluate the presence of a gradient in the amount of generated anterograde interference, which would be prevented by the dichotomous interpretation of *p* values (Ho et al., 2019).

Concerning Hand Direction at PV, the results revealed a Conditions * Sessions * Phases interaction (*F*_(6,138)_ = 2.423, *p* = .029, $\eta_{p}^{2}=.095$), indicating that participants behaved differently across Sessions and Phases across the four Conditions. This interaction is decomposed below.

### 2.1 Anterograde interference scales as a function of task similarity

Regarding B → A (see Figure 2a), the results revealed a Sessions * Phases interaction (*F*_(2,46)_ = 9.463, *p* < .001, $\eta_{p}^{2}=.292$). Pairwise comparisons revealed that the second Baseline, Acquisition and Retention phases were, respectively, lower than the first Baseline (*t*_(23)_ = 4.749, *p* < .001, Cohen's *d*_z_ = .969 [.475 1.450]), Acquisition (*t*_(23)_ = 2.738, *p* = .012, Cohen's *d*_z_ = .559 [.123 .985]) and Retention phases (Wilcoxon's *W* = 300.0, *p* < .001, Cohen's *d*_z_ = 1.392 [.818 1.949]). These results suggest that initial learning of B induced a *negative* reach bias at Baseline and impaired adaptation levels in both Acquisition and Retention of A during the second session (Hinder et al., 2007; Lerner et al., 2020).

Regarding A → A (see Figure 2b), the results revealed a Sessions * Phases interaction (*F*_(2,46)_ = 13.513, *p* < .001, $\eta_{p}^{2}=.370$). Pairwise comparisons revealed that while adaptation levels were greater in the second Baseline as compared with the first one (*t*_(23)_ = 3.046, *p* = .009, Cohen's *d*_z_ = .622 [.178 1.054]), adaptation levels were similar during Acquisition (*t*_(23)_ = .385, *p* = .704, Cohen's *d*_z_ = .079 [−.323 .478]) and were lower during the second Retention phase as compared with the first one (*t*_(23)_ = 3.337, *p* = .004, Cohen's *d*_z_ = .681 [.230 1.121]). These results suggest that initial learning of A induced a *positive* reach bias at Baseline did not facilitate subsequent Acquisition (no apparent savings) and impaired Retention of the second A. This fully replicates previous findings (Hamel et al., 2021).

Importantly, an additional pairwise comparison confirmed that there was an important difference (i.e., above a large effect size) between the Baseline of the second session in the B → A condition and the Baseline of the second session in the A → A condition (*t*_(23)_ = 9.805, *p* < .001, Cohen's *d*_z_ = 2.001 [1.294 2.694]). This suggests that the large differences in baseline reach biases between the two conditions must be accounted for before a direct comparison of the amount of generated anterograde interference can be made (see below).

Regarding A′ → A (see Figure 2c), the results revealed a marginal Sessions * Phases interaction (*F*_(2,46)_ = 3.139, *p* = .065, $\eta_{p}^{2}=.120$), which was explored. Pairwise comparisons revealed that both the second Baseline (*t*_(23)_ = .552, *p* = .586, Cohen's *d*_z_ = .113 [−.290 .513]) and Acquisition phases (*t*_(23)_ = 1.273, *p* = .324, Cohen's *d*_z_ = .260 [−.150 .664]) did not meaningfully differ between the two sessions. Interestingly, Retention was modestly lower in the second session as compared with the first one (*t*_(23)_ = 2.007, *uncorrected p* = .057, Cohen's *d*_z_ = .410 [−.011 .823]), which suggests that the marginal interaction was driven by the modest Retention impairment. However, this comparison did not survive the correction for multiple comparisons (*corrected p* = .171). This nonetheless suggests that modifying the reaching movement direction upon the initial learning of A′ generated modest anterograde interference upon subsequent learning of A.

Regarding α → A (see Figure 2d), the results neither revealed a Sessions * Phases interaction (*F*_(2,46)_ = .379, *p* = .621, $\eta_{p}^{2}=.016$) nor an effect of Sessions (*F*_(1,23)_ = 1.854, *p* = .187, $\eta_{p}^{2}=.075$), suggesting that initial left‐handed learning of α did not meaningfully interfere with subsequent right‐handed learning of A. This also suggests that cognitive fatigue did not accumulate and thus cannot account for the anterograde interference effect observed in the other conditions. For the effect size values of each pairwise comparison, see the captions of Figure 2.

Altogether, these results indicate that anterograde interference emerges along a gradient determined by the similarity between the tasks; the more similar the two learning tasks occurring in close temporal proximity are (as in B → A and A → A), the more anterograde interference is generated between them (see Figure 4a). Segregating the neural networks involved in the two learning sessions entailed modest (A′ → A) and negligible (α → A) amounts of anterograde interference. The quantitative support to the presence of such a gradient comes from the effect sizes of the Retention differences, which gradually decreased from the B → A (Cohen's *d*_z_ = 1.392), A → A (Cohen's *d*_z_ = .681), A′ → A (Cohen's *d*_z_ = .410) to the α → A conditions (Cohen's *d*_z_ = .267).

### 2.2 Similarly impaired learning capabilities in both B → A and A → A when accounting for baseline reach biases

Baseline priors can confound the assessment of anterograde interference (Lerner et al., 2020; Sing & Smith, 2010). To establish the degree to which each condition impaired learning and retention capabilities, it is crucial to account for potential differences observed at Baseline in both the first and second sessions. Two separate RM ANOVAs conducted on Hand Direction at PV data revealed no meaningful effect of Conditions at the Baseline of the first session (*F*_(3,69)_ = .8616, *p* = .465, $\eta_{p}^{2}=.036$) but revealed important differences at the Baseline of the second session (*F*_(3,69)_ = 11.729, *p* < .001, $\eta_{p}^{2}=.338$). Pairwise comparisons revealed that the performance levels of the second Baseline differed between every condition (all *t*_(23)_ > 2.684, all *p* < .016, all Cohen's *d*_z_ > .548 [.113 .973]), except between the A′ → A and α → A conditions (*t*_(23)_ = .324, *p* = .749, Cohen's *d*_z_ = .066, [−.335 .466]). These results indicate that performance levels did not differ upon the start of the first session, thus arguing against between‐condition carryover effects. Importantly, they also reveal that adaptation occurring during the first session conditioned the Baseline performance levels in the second one, justifying the need to control for these differences before the extent of impairment in learning and retention capabilities across conditions can be evaluated.

To account for the important differences of the second Baseline, for Hand Direction at PV data only, each session's average Baseline data were subtracted from the corresponding session's average Acquisition and Retention data. This was done for each participant and each condition. Data were then submitted to a 4 Conditions * 2 Sessions * 2 Phases (Acquisition, Retention) RM ANOVA. The results revealed a Conditions * Sessions * Phases interaction (*F*_(3,69)_ = 3.067, *p* = .034, $\eta_{p}^{2}=.118$), which was decomposed by conducting separate Sessions * Phases RM ANOVAs for each condition.

**FIGURE 3:**
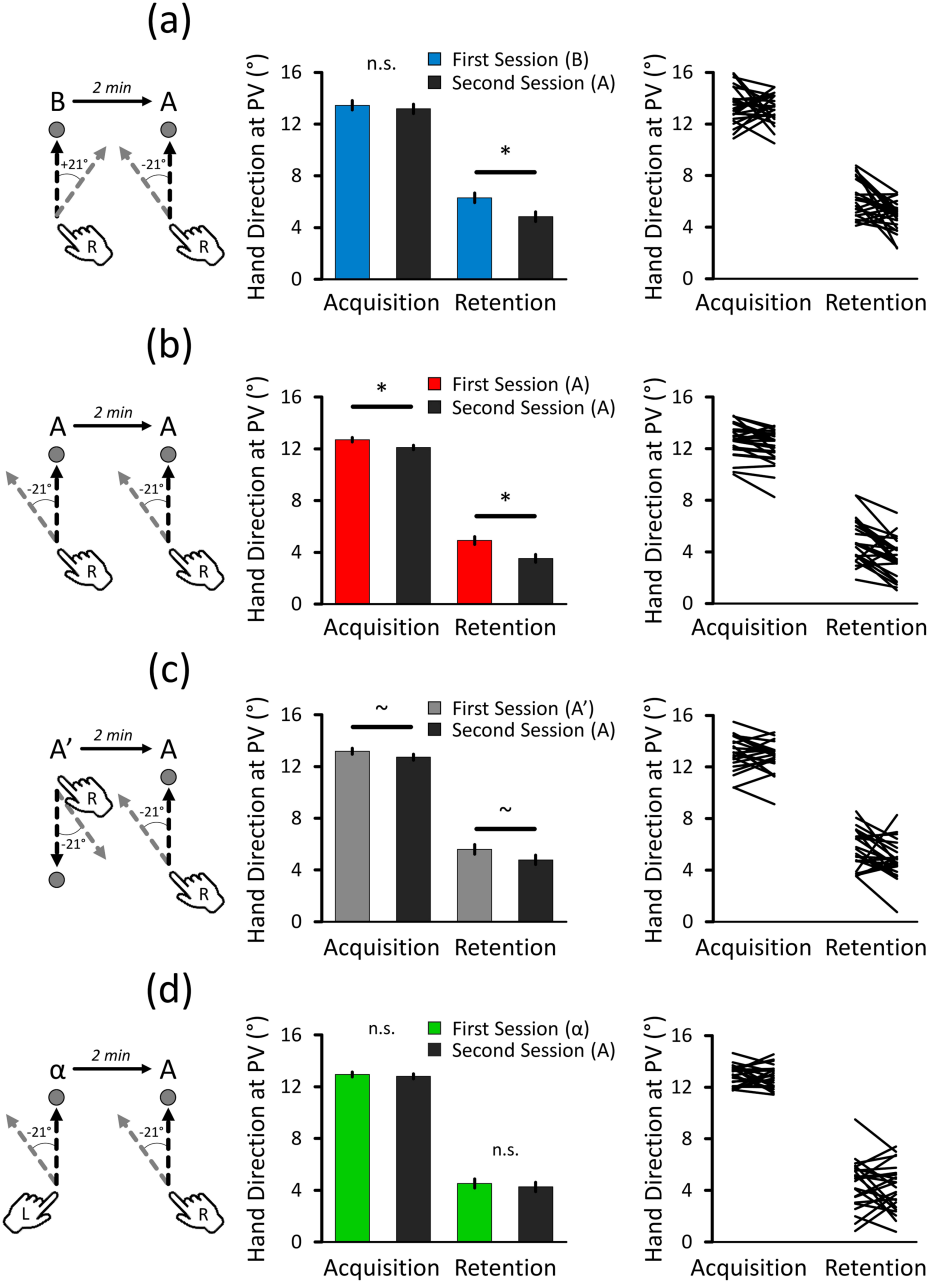
Baseline‐corrected Hand Direction at peak velocity (PV) data. The leftmost column depicts the procedures of each condition while the rightmost column represents the individual data contained in the adjacent bar graphs. (a) B → A condition. Correcting for prior differences in Baseline reach biases revealed that subsequent Acquisition was no longer impaired (*d*_z_ = .148 [−.256 .549]) whereas Retention remained impaired (*d*_z_ = .793 [.326 1.247]). (b) A → A condition. Correcting for prior difference at Baseline revealed that both subsequent Acquisition (*d*_z_ = .722 [.265 1.167]) and Retention (*d*_z_ = .921 [.434 1.393]) were meaningfully impaired. Note that additional results revealed that both the Acquisition and Retention adaptation level differences did not differ between the B → A and A → A conditions, suggesting similarly impaired learning and retention capabilities. (c) A′ → A condition. The results revealed a main effect of Sessions, which indicated that both the subsequent Acquisition (*d*_z_ = .397 [−.022 .809]) and Retention (*d*_z_ = .444 [.019 .859]) were modestly impaired. (d) α → A condition. The results revealed no meaningful difference in either Acquisition (*d*_z_ = .143 [−.261 .543]) or Retention (*d*_z_ = .150 [−.254 .551]). Asterisks (*) and tildes (~) represent significant and marginal differences, respectively. For all panels, the mean ± 95% within‐subject confidence intervals are depicted

Regarding B → A (see Figure 3a), the results revealed a Sessions * Phases interaction (*F*_(1,23)_ = 13.777, *p* = .001, $\eta_{p}^{2}=.375$). Pairwise comparisons revealed that Acquisition phases were no longer meaningfully different (*t*_(23)_ = .727, *p* = .475, Cohen's *d*_z_ = .148 [−.256 .549]), but that Retention remained largely impaired in the second session as compared with the first one (*t*_(23)_ = 3.887, *p* = .001, Cohen's *d*_z_ = .793 [.326 1.247]). This suggests that the differences in Baseline reach biases in the B → A condition explained the observed interference during the Acquisition phase but not in the Retention phase.

Regarding A → A (see Figure 3b), the results revealed a Sessions * Phases interaction (*F*_(1,23)_ = 6.765, *p* = .016, $\eta_{p}^{2}=.227$). Pairwise comparisons revealed that both the Acquisition (*t*_(23)_ = 3.537, *p* = .002, Cohen's *d*_z_ = .722 [.265 1.167]) and Retention phases (*t*_(23)_ = 4.511, *p* < .001, Cohen's *d*_z_ = .921 [.434 1.394]) were meaningfully impaired in the second session as compared with the first one. This shows that learning the same task twice meaningfully impaired both learning and retention capabilities upon the second session, even when accounting for baseline reach biases.

To determine if learning and retention capabilities were similarly impaired in both the B → A and A → A conditions, the difference between the first and second sessions of each phase and each condition was submitted to a 2 Conditions * 2 Phases (Acquisition difference, Retention difference) RM ANOVA. The results revealed no Conditions * Phases interaction (*F*_(1,23)_ = .892, *p* = .355, $\eta_{p}^{2}=.037$) and no effect of Conditions (*F*_(1,23)_ = .113, *p* = .740, $\eta_{p}^{2}=.005$). This suggests that despite the above non‐significant difference at Acquisition in the B → A condition, learning (Cohen's *d*_z_ = .201 [−.206 .603]) and retention capabilities (Cohen's *d*_z_ = .026 [−.374 .426]) were similarly impaired in both the B → A and A → A conditions. These results suggest that mechanisms other than competing memories also contribute to anterograde interference and emphasize that differences in Baseline reach biases can confound the assessment of anterograde interference (Lerner et al., 2020; Sing & Smith, 2010).

Regarding A′ → A (see Figure 3c), the results did not reveal a Sessions * Phases interaction (*F*_(1,23)_ = 1.057, *p* = .315, $\eta_{p}^{2}=.044$) but revealed an effect of Sessions (*F*_(1,23)_ = 6.059, *p* = .022, $\eta_{p}^{2}=.209$), which indicated that average levels of adaptation were lower in the second session (8.75° ± .25°) as compared with the first one (9.36° ± .25°). Pairwise comparisons across Phases were conducted to confirm that the effect of Sessions was present in both the Acquisition and Retention phases. Namely, the results revealed that both Acquisition (*t*_(23)_ = 1.947, *p* = .064, Cohen's *d*_z_ = .397 [−.023 .809]) and Retention phases (*t*_(23)_ = 2.174, *p* = .081, Cohen's *d*_z_ = .444 [.019 .859]) were modestly impaired. Reinforcing the above results, this indicates that decreasing tasks' similarity—likely by segregating the directionally tuned neural populations involved in both sessions (Cowper‐Smith et al., 2010; Eisenberg et al., 2010; Fabbri et al., 2010; Haar et al., 2015; Mahan & Georgopoulos, 2013; Tanaka et al., 2018; Toxopeus et al., 2011)—decreases the degree of anterograde interference between two tasks (Crossley et al., 2019).

Regarding α → A (see Figure 3d), the results neither revealed a Sessions * Phases interaction (*F*_(1,23)_ = .155, *p* = .698, $\eta_{p}^{2}=.007$) nor an effect of Sessions (*F*_(1,23)_ = .710, *p* = .408, $\eta_{p}^{2}=.030$), confirming that initial left‐handed learning of α did not meaningfully interfere with the subsequent right‐handed learning of A. This indicates that further decreasing tasks' similarity—by using different limbs to maximally segregate the learning‐specific neural networks—meaningfully lessens the degree of generated anterograde interference (Crossley et al., 2019). For the effect size values of each pairwise comparison, see the captions of Figure 3.

### 2.3 No evidence of fatigue accumulation between the two learning sessions

Readers are referred to the Supporting Information for evidence of the absence of fatigue accumulation between the two learning sessions, as results revealed either an improvement or stability in all RT, endpoint accuracy, MT and hit rates across sessions.

## 3 DISCUSSION

**FIGURE 4:**
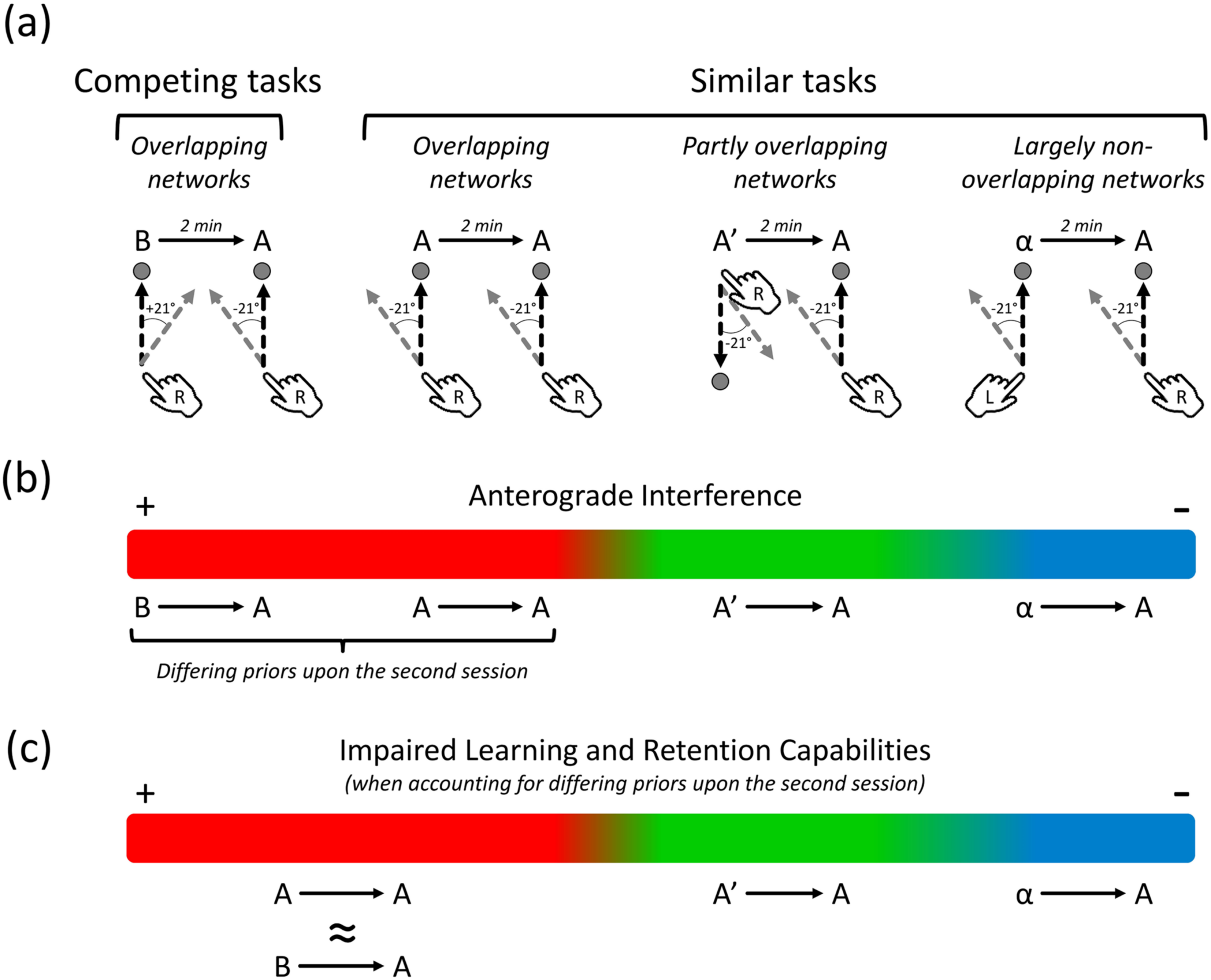
Conceptual representation of the methods and results. (a) Schematic representation of the rationale and methodological procedures. (b) Interpretation of the results from the non‐baseline‐corrected data (Figure 2). The B → A, A → A, A′ → A and α → A conditions generated large (Cohen's *d*_z_ of ~1.4), meaningful (Cohen's *d*_z_ of ~.7), modest (Cohen's *d*_z_ of ~.4) and negligible (Cohen's *d*_z_ of ~.2) amounts retention impairments. This indicates that anterograde interference was generated along a gradient as a function of the present experimental manipulations. To determine if the B → A and the A → A showed similarly impaired learning capabilities, the important differences (Cohen's *d*_z_ of ~2.0) between the Baseline of the second session of each condition had to be accounted for. (c) Interpretation of the results from the baseline‐corrected data (Figure 3). The B → A and A → A conditions showed similarly impaired learning and retention capabilities (Cohen's *d*_z_ of ~.8), while the A → A and α → A conditions generated modest (Cohen's *d*_z_ of ~.4) and negligible (Cohen's *d*_z_ of ~.2) amounts of anterograde interference, respectively. Altogether, these results suggest that learning‐specific neural networks must be maximally dissociated to minimize the interfering influence of previous learning on subsequent learning

This work tested the hypothesis that anterograde interference emerges along a gradient according to the putative degree of overlap between learning‐specific neural networks; learning similar tasks, most likely by recruiting overlapping neural networks, should generate more anterograde interference than learning dissimilar tasks. The results fully supported this hypothesis by revealing gradually decreasing effect sizes in the Retention impairments across conditions. Namely, the novelty of this work is that the results revealed decreasing amounts of anterograde interference from the A → A (Cohen's *d*_z_ of ~.7; meaningful effect size), A′ → A (Cohen's *d*_z_ of ~.4; modest effect size), to α → A conditions (Cohen's *d*_z_ of ~.2; negligible effect size), that is as a function of the putative decrease in the overlap between the neural networks recruited in the two learning sessions (see Figure 4). Moreover, the results also revealed that the B → A and A → A conditions generated similarly impaired learning and retention capabilities, which is consistent with previous results indicating that memory competition is not required to induce anterograde interference (Hamel et al., 2021). One enticing possibility is that initial learning, by perturbing the homeostasis of learning‐specific networks, impairs neuroplastic capabilities in its associated neural network. If subsequent learning recruits a sufficiently overlapping neural network, then anterograde interference emerges (Crossley et al., 2019).

### 3.1 Competing motor memories may not be required to generate anterograde interference

One important novel result of this work stems from the direct comparison of the B → A and A → A conditions. The present results revealed that the initial learning of B interfered with the subsequent acquisition and retention of A, which is consistent with previous work (Hinder et al., 2007; Lerner et al., 2020). However, the results also revealed that initial learning of A interfered with the subsequent retention of A, which fully replicates previous findings (Hamel et al., 2021) and suggests that anterograde interference can also emerge even if motor memory competition is prevented from occurring by learning the same task twice. Moreover, the results revealed important differences (i.e., Cohen's *d*_z_ of ~2.0; above‐large effect size) between the Baseline adaptation levels upon the second session of the B → A and A → A conditions. Namely, the ostensibly greater levels of impairment in the B → A as compared with the A → A condition may have stemmed from reaching priors in a direction opposite (~−1.5°) to the one required for subsequent adaptation (towards +21°), whereas in the A → A condition, the reaching priors were in the same direction (~+.4°) as subsequent adaptation (towards +21°). Given that such differences can confound the evaluation of anterograde interference (Lerner et al., 2020; Sing & Smith, 2010), the Baseline adaptation levels were subtracted from the Acquisition and Retention adaptation levels in subsequent analyses. These results revealed that the Acquisition (Cohen's *d*_z_ of ~.2; negligible to small effect size) and Retention adaptation levels (Cohen's *d*_z_ below .1; negligible effect size) were similarly impaired in both the B → A and A → A conditions. Together, these results support the presence of anterograde interference in B → A paradigms but extend the memory competition interpretation (Herszage & Censor, 2018; Robertson, 2018) by suggesting that mechanisms other than—or in addition to—competing memories also constrain subsequent learning and retention capabilities and thus contribute to the emergence of interference (see below).

One may wonder if the present retention impairments could also be observed when using different motor tasks. Interestingly, recent work suggests that the present retention impairments are not specific to the present experimental procedures. On the one hand, Branscheidt et al. (2019) recently showed that the accumulation of central fatigue can disrupt both learning and long‐term memory of a novel pinch motor skill, suggesting that the present results transfer to different motor tasks and that the present immediate retention impairments are long‐lasting. On the other hand, given that synaptic plasticity mediates learning (Magee & Grienberger, 2020) and that biological constraints emerge to regulate synaptic plasticity and preserve homeostasis (Li et al., 2019; Yee et al., 2017), it appears reasonable to assume that virtually every form of learning could be altered by the emergence of homeostatic constraints (Kukushkin & Carew, 2017; Lee & Kirkwood, 2019). Altogether, these results suggest that the present retention impairments would generalize to other motor tasks. Until confirmed by future studies, this possibility should remain tentative.

### 3.2 Putatively overlapping neural networks may be required for anterograde interference to emerge

One important result is that the initial learning of A (−21°; right arm, upper targets), A′ (−21°; right arm, lower targets) and α (−21°; left arm, upper targets) meaningfully, modestly and negligibly impaired retention upon the subsequent learning of A (−21°; right arm, upper targets), that is even if similar memories were systematically acquired. The novelty of these results resides in the presence of a gradient in the retention impairments, which is quantitatively supported by the effect sizes of the Retention differences across conditions: A → A (Cohen's *d*_z_ of ~.7; meaningful effect size), A′ → A (Cohen's *d*_z_ of ~.4; modest effect size) and α → A (Cohen's *d*_z_ of ~.2; negligible effect size). Similar results were found even after correcting for differences in baseline reach biases, which suggests that these retention impairments were not a by‐product of differing priors. Importantly, neuroimaging and behavioural results indicate that modifying reach direction (Mahan & Georgopoulos, 2013; Schween et al., 2018) and the effector used (Bernier et al., 2012; Werner et al., 2019) recruits distinct neuronal networks. These lines of evidence imply that learning‐specific neural networks overlapped importantly in the A → A condition because of identical reaching conditions, moderately in the A′ → A condition because of a change in reach direction and minimally in the α → A condition because of a change in the used effector. Assuming that such a gradient in the learning‐specific neural networks across conditions occurred in the present work, then one inference is that the retention impairments emerged along a gradient as a function of the learning sessions' similarity: learning under similar reaching conditions (A → A)—likely by recruiting overlapping neural networks and thus prompting the emergence of homeostatic constraints—generated more interference than learning under dissimilar reaching conditions (α → A). The notion of a gradient is an important one; just as there can never be zero effect in null hypothesis testing (Greenland, 2019; Lakens, 2017), anterograde interference is most likely never completely absent when two learning sessions—even if vastly different ones—occur in close temporal proximity (Keisler & Shadmehr, 2010; Kim, 2020). The presence of a gradient in the present results also argues against the possibility that the Washout phase, by prompting performance to return towards Baseline levels—acted as a competing memory (Hinder et al., 2007); if the presence of a Washout accounted for the present results, then similar amounts of anterograde interference would have been present in every condition and would have thus not emerged along a gradient. The present gradient also argues against the possibility that the accumulation of fatigue over trials—whether cognitive or physical—could account for the present results, as all sessions contained the same number of trials. The results further revealed that RTs quickened, MT and hit rates remained stable and endpoint accuracy improved from the first to the second session (see the Supporting Information), further arguing against the possibility that fatigue accumulation could account for the present gradient of anterograde interference. Globally, based on the present behavioural manipulations that sought to manipulate the putative overlap between the neural networks of A, A′ and α, one compelling interpretation of the present results is that overlapping neural networks are required for anterograde interference to emerge (Crossley et al., 2019).

Recent human studies echo the present interpretation by showing that the provision of contextual cues allows one to concomitantly learn otherwise opposing tasks with minimal interference (Hirashima & Nozaki, 2012; Howard et al., 2015; Sheahan et al., 2016). Namely, Howard et al. (2015) and Sheahan et al. (2016) have shown that the cued planning of distinct follow‐through movements associated with either a clockwise or counterclockwise force‐field (FF) exposure allows participants to adapt to both opposite deviations as compared with conditions where no follow‐through movements were to be planned. The authors proposed that follow‐through movement planning differentiated the neural networks mediating FF adaptation, therefore permitting the concomitant learning of both opposite perturbations with minimal interference (Howard et al., 2015; Sheahan et al., 2016). Here, the experimental manipulations sought to achieve a similar goal—to minimize the emergence of anterograde interference by differentiating learning‐specific neural networks—but by changing the parameters of the reaching instead of the planning conditions. Overall, the above along with the present results suggest that manipulations dissociating learning‐specific neural networks where two tasks are learned in close temporal proximity efficiently minimize the emergence of interference. Other means to minimize interference include limiting the amount of initial learning and allowing a sufficient amount of time (>1 h) between the learning of two tasks (Hamel et al., 2021; Lerner et al., 2020). Repetitively practicing the interfering learning conditions over separate days (e.g., Yotsumoto et al., 2013) could also provide a means to afford resistance to—and enable high‐performance levels in the presence of—anterograde interference (see Strobach & Torsten, 2017).

### 3.3 Mechanistic account for the observed gradient in anterograde interference

From a neurobiological perspective, the notion of memory competition has been argued to emerge on the basis that initial learning induces a shortage of learning‐critical biological resources, such as synaptic plasticity‐related proteins (Herszage & Censor, 2018), therefore interfering with subsequent learning. However, the present results warrant the refining of this interpretation, because they indicate that memory competition is not the only contributor to interference. While such a network‐specific shortage of biological resources could constitute a negative feedback loop constraining subsequent learning capabilities, this interpretation neglects an extensive literature showing that additional biological mechanisms constrain learning and memory (Kukushkin & Carew, 2017; Li et al., 2019; Moreno, 2020; Smolen et al., 2016; Yee et al., 2017). Specifically, the homeostatic plasticity framework posits that initial learning—by incurring synaptic plasticity—quickly perturbs neuronal metabolic homeostasis (Yee et al., 2017). To preserve homeostasis, biological constraints quickly emerge to prevent further deviations from homeostasis by limiting subsequent learning capabilities (Keck, Toyoizumi, et al., 2017). This framework differs from the memory competition interpretation, as it does not necessarily imply a shortage of biological resources for interference to emerge. Upon initial learning, biological mechanisms including the modification of NMDA receptor activity (Zorumski & Izumi, 2012), the activation of protein phosphatases (Genoux et al., 2002; Moreno, 2020) or the release of adenosine in the extracellular medium (Dias et al., 2013) could emerge to constrain and interfere with subsequent learning capabilities. Although speculative, the present results nonetheless suggest that biological mechanisms other than—or in addition to—competing memories contribute to the emergence of anterograde interference.

If it can be assumed that the homeostatic plasticity framework viably allows interpreting the present findings, then other implications would follow. Namely, the documented biological mechanisms shown to constrain synaptic plasticity are mostly molecular and cellular (Lee & Kirkwood, 2019), which presumably renders the homeostatic constraints largely circuit and neuron specific (reviewed in Kukushkin & Carew, 2017; Moreno, 2020; Smolen et al., 2016). As argued above, previous works (Branscheidt et al., 2019; Hirashima & Nozaki, 2012; Howard et al., 2015; Sheahan et al., 2016), as well as the present results, support the inference that such constraints are network‐specific.

If the known homeostatic constraints are presumably circuit and neuron specific (Kukushkin & Carew, 2017; Lee & Kirkwood, 2019; Smolen et al., 2016), then how can putatively segregated neural networks still generate evidence of modest (Cohen's *d*_z_ of ~.4) and negligible (Cohen's *d*_z_ of ~.2) anterograde interference in the A′ → A and α → A conditions, respectively? A first possibility is that the task‐specific neural networks were indeed non‐overlapping but the activity of the first network interfered with the neuroplasticity induction capabilities of the second network via the paracrine releasing of extracellular adenosine (see Dias et al. (2013) for a review). Adenosine—a metabolite of ATP consumption—is regarded as a molecule that conditions the brain's learning capabilities as a function of its past neural activity (Dias et al., 2013). Once released, adenosine inhibits excitatory synaptic transmission (Yamashiro & Morita, 2017) and shifts synaptic plasticity regimes from Hebbian to homeostatic (Bannon et al., 2017). By increasing its ATP consumption, a neural network incurring learning‐dependent synaptic plasticity would release adenosine in the extracellular space (Dias et al., 2013), therefore interfering with the induction of synaptic plasticity in its own and neighbouring neural networks (Bannon et al., 2017; Dias et al., 2013). Future studies could administer caffeine (an adenosine receptor antagonist; Ribeiro & Sebastião, 2010) to investigate its contribution to the emergence of anterograde interference.

A second possibility is that portions of the learning‐recruited neural networks nonetheless overlapped to modest and negligible degrees in the A′ → A and α → A conditions, respectively, which consequently generated modest and negligible levels of anterograde interference. Correspondingly, recent neuroimaging work showed reach direction‐*independent* preparatory activity in the primate motor and dorsal premotor cortices (Kaufman et al., 2016) and effector‐*independent* recruitment of human frontoparietal associative areas during movement execution (Haar et al., 2017; Liu et al., 2020). These results suggest that manipulating reach direction or the effector used during learning may still recruit partly overlapping neural networks, albeit less so than when tasks are identical. Moreover, Kumar et al. (2018) reported evidence of retrograde (but not anterograde) interference when learning abrupt opposite sensorimotor perturbations with the right and left arms (see Stockinger et al. (2017) for similar results). However, previous work indicated such between‐limb interference may not occur when the perturbations are learned gradually (as in the present paradigm) rather than abruptly (Malfait & Ostry, 2004; Werner et al., 2019) (but see Wang et al., 2011), which makes it uncertain how much the results of Kumar et al. (2018) translate to the present work. Nonetheless, the above lines of evidence suggest that the neural networks could have partly overlapped, thus impairing neuroplasticity induction capabilities in the brain areas where networks overlapped and generating modest (Cohen's *d*_z_ of ~.4) and negligible (Cohen's *d*_z_ of ~.2) anterograde interference in the present A′ → A and α → A conditions, respectively. Further work is warranted to confirm these possibilities.

### 3.4 Testable predictions for future work

If the present results can be interpreted in light of the homeostatic plasticity framework, then it becomes tempting to infer that other phenomena known to facilitate the emergence of homeostatic constraints should further amplify the amount of generated anterograde interference. One testable prediction comes from the synaptic homeostasis hypothesis (SYH), an influential model that posits that neural networks almost exclusively incur synaptic potentiation during wakefulness (Tononi & Cirelli, 2020). This extended synaptic potentiation prompts homeostatic constraints to gradually increase as the day progresses (Fernandes & Carvalho, 2016), which would consequently increase the amount of anterograde interference. Based on the SYH, a resulting hypothesis is that the amount of anterograde interference becomes increasingly important over a wake period (Lehr et al., 2021); if confirmed by future studies, this would further support the interpretation that homeostatic constraints drive anterograde interference. Importantly, here, participants were systematically tested at the same time of day, which makes it unlikely that these considerations can bias the interpretation of the present results. Finally, whether the time of day influences human learning and memory remains to be ascertained (see de Beukelaar et al., 2016; Sale et al., 2013) before an inference can be drawn to anterograde interference.

## 4 LIMITATIONS OF THE STUDY

One limitation of the present study is the lack of direct neural data to support the presumed segregation of the neural networks. However, the assumptions made on the recruited neural networks rest on extensive and converging lines of work showing that (1) the learning tasks A and B in a short time period induces interference because of the overlap in their putative neural networks (Herszage & Censor, 2018), (2) modifying reach direction recruits distinct directionally tuned neurons confined to the same hemisphere (Cowper‐Smith et al., 2010; Eisenberg et al., 2010; Fabbri et al., 2010; Haar et al., 2015; Mahan & Georgopoulos, 2013; Tanaka et al., 2018; Toxopeus et al., 2011) and (3) limb‐evoked neural activity is largely lateralized (Bernier et al., 2012; Chang et al., 2008; Gallivan et al., 2011; Levy, 1969; Serrien et al., 2006; Welniarz et al., 2015; Yttri et al., 2014). This evidence is not incompatible with neuroimaging work showing different degrees of overlap in the neural networks involved in task‐switching paradigms (Bode & Haynes, 2009; Brass & von Cramon, 2004; De Baene & Brass, 2011; Jiang, 2004; Wylie et al., 2004, 2006), suggesting that manipulating low‐level task features effectively enables to segregate—at least partially—neural networks. Although this evidence suggests that the recruited neural networks were segregated along a gradient in the present study, this support remains correlational and warrants confirmation by future studies.

A second matter that could be seen as a limitation is the use of a within‐subject learning design, as it could yield carryover effects from one condition to the next that could confound the interpretation of the present results. However, condition ordering was fully counterbalanced across participants (4! Conditions means 24 possible condition orderings). This implies that each of the 24 participants had a unique condition ordering, therefore averaging out a potential carryover effect across conditions (Brooks, 2012). Moreover, the potential residual effect of the preceding session was washed out with a lengthy Familiarization block for both the right and left arms before the start of each experimental session (Brooks, 2012). Results confirmed the absence of a carryover effect at the Baseline of the first session across experimental conditions, suggesting that a carryover effect does not confound the present results. The use of a fully within‐subject design was also meant to optimize statistical power to improve the reliability of the results reported (Algermissen & Mehler, 2018; Button et al., 2013).

## 5 METHODS

Most of the materials and methods are identical to the ones used in Hamel et al. (2021). Readers are thus referred to the methods of Hamel et al. (2021) and the Supporting Information for further methodological details.

### 5.1 Participants

A total of 24 healthy right‐handed human participants took part in this study (12 females; 23.5 ± .9 years old; all reported values represent means ± 95% confidence intervals). The sample size was determined by the experimental design. Namely, to fully counterbalance the four within‐subject conditions (Figure 1), 24 participants were required (4! possible condition ordering). A sensitivity analysis (G*Power v3.9.1.2) revealed that 24 participants allowed detecting significant differences with a mean effect size (Cohen's *d*_z_) of .597 at 80% power when using two‐tailed dependent *t* tests. In comparison, an a priori power analysis using the same parameters revealed that two groups of 46 individuals each are required to achieve the same effect size in a between‐subject design when using two‐tailed independent *t* tests.

Participants were self‐reported neurologically healthy with normal or corrected‐to‐normal vision. Informed consent forms approved by the ethical committee of the Centre intégré universitaire de santé et services sociaux de l'Estrie were signed before the start of the experiment. The experiment conformed to the standards set by the Declaration of Helsinki.

### 5.2 Gradual visuomotor adaptation paradigm

In a virtual environment, participants had to perform 10‐cm‐long centre‐out reaching movements with their right hand towards one of three visual targets while adapting to a visual deviation. When located in the upper quadrants, the three targets were positioned at 45°, 90° and 135°. When located in the lower quadrants, the three targets were positioned at 225°, 270° and 315°. The time course of visuomotor adaptation per condition and session is shown in Figure 1. All sessions were identical in terms of go–cue delay, target order presentation and trial‐per‐trial delivery of performance‐contingent feedback.

Before the start of the experiment, a practice phase of 135 trials preceded the first session to allow participants to familiarize themselves with the task requirements (Familiarization; not shown in Figure 1). Namely, participants first familiarized with right‐handed reaching movements towards one of three targets located in the upper quadrants (45 trials; A and B conditions), then with right‐handed reaching movements towards one of three targets located in the lower quadrants (45 trials; A′ condition) and, finally, with left‐handed reaching movements towards one of three targets located in the upper quadrants (45 trials; α condition). These data were used to adjust targets' width to homogenize hit rates across individuals and conditions (see the Supporting Information) and to account for the effector‐ and direction‐dependent reach biases (see below).

The following procedures are shown in Figure 1. Following Familiarization, participants first executed a Baseline phase (60 trials) with veridical visual feedback. For the A, A′ and α conditions, participants adapted to a gradually introduced visual deviation that steadily increased by −.7° per three‐trial bins (cycles) over 90 trials (Ramp phase) until it reached −21°. Then, the visual deviation was maintained constant at −21° for 90 trials (Hold phase). For the B condition, the gradual introduction of the visual deviation was identical to the A, A′ and α conditions, but the sign of the deviation was inverted (+21° rather than −21°). Together, the Ramp and Hold phases are hereafter referred to as the Acquisition phase.

Immediately upon completion of the Acquisition phase, reaching aftereffects were assessed with an initial phase in which vision of the cursor was occluded for 60 trials (NoVision phase). This phase allowed evaluating the persistence of the adapted reaching behaviours in the absence of corrective visual feedback. Subsequently, participants executed 60 trials with veridical visual feedback (Washout phase). Together, the NoVision and Washout phases are hereafter referred to as the Retention phases. Participants were never informed that a visual deviation had been introduced.

### 5.3 Accounting for the condition‐specific reach biases observed during familiarization

During familiarization, participants showed differences in initial reach biases among the different reaching conditions (see the Supporting Information). These are most likely due to the different biomechanical constraints of reaching with different limbs (left vs. right arm) and to different quadrants when using the right arm (upwards vs. downwards; McCrea et al., 2002). To ensure that these differences would not bias the analysis of Hand Direction at PV during the learning sessions, the average of each reach condition during familiarization (right arm to upper targets, right arm to lower targets, left arm to upper targets) was drawn across all four experimental visits and was then subtracted to its associated learning session for each participant. Specifically, the average Hand Direction at PV of the first 45 familiarization trials (right arm, upper targets) was used to normalize the B and A conditions, which involved right‐handed reaching movements to upper targets. The average Hand Direction at PV of the subsequent 45 familiarization trials (right arm, lower targets) was used to normalize the A′ condition, which involved right‐handed reaching movements to lower targets. Finally, the average Hand Direction at PV of the last 45 familiarization trials (left arm, upper targets) was used to normalize the α condition (left arm, upper targets), which involved left‐handed reaching movements to upper targets.

Because a +21° visual deviation was learned in B and a −21° visual deviation was learned in A, the Hand Direction at PV data were of different polarity. To properly compare the B → A and A → A conditions, the Acquisition and Retention average Hand Direction at PV data—but not the Baseline data—were injected as absolute values in the statistical analyses. The Baseline data were not included as absolute values in the analyses because no visual deviation was implemented in this phase. The same procedure was applied to the data of the B session shown in Figure 2; namely, apart from Baseline, all data points included in the Acquisition and Retention represent absolute Hand Direction at PV values.

### 5.4 Definition of the dependent variables of interest

A custom‐made MATLAB script was used to display and acquire kinematic data during the experiment. The primary variable of interest was Hand Direction at peak tangential velocity (PV), which was used to evaluate performance. This early kinematic marker was chosen because it is considered a reflection of the movement planning process (Carlton, 1992). Additionally, RT (defined as the temporal difference in milliseconds between the auditory Go cue and movement onset), endpoint accuracy (the absolute distance in centimetres between the cursor and target centroids at movement end), MT (defined as the temporal difference in milliseconds between movement onset and movement end) and hit rates (defined as the binarization of task success based on endpoint accuracy) were also analysed. For information on how individual differences in hit rates were accounted for, see the Supporting Information. Data were averaged across phases (Baseline, Acquisition, Retention) to perform subsequent analyses.

### 5.5 Outlying data rejection

Outlying trials were detected based on RT, MT and endpoint accuracy data. Namely, individual trials were excluded from all analyses if RTs were below 100 ms or above 3 median absolute deviations (MADs; Leys et al., 2013) or if MTs were ±3 MAD from each participant's median. Accuracy at movement endpoint greater than 10 cm also resulted in trial rejection.

## CONFLICT OF INTEREST

The authors declare no competing financial interests.

## AUTHOR CONTRIBUTIONS

R.H. designed the experiment, collected the data, conducted the analyses, prepared the figures and wrote the manuscript. J.F.L. and P.M.B. revised the manuscript.

### PEER REVIEW

The peer review history for this article is available at https://publons.com/publon/10.1111/ejn.15561.

## Supporting information


**Data S1.** Supporting Information

## Data Availability

This work's data are freely available at the following URL: https://drive.google.com/drive/folders/1IdKIal1dKa21W_asWLisVncFMew9XuaN?usp=sharing.
