# Supplementary material for: Anterograde interference emerges along a gradient as a function of task similarity: A behavioural study
Source: Eur J Neurosci. 2021 Dec 20;55(1):49–66. doi: 10.1111/ejn.15561 (PMC9299670; doi:10.1111/ejn.15561)
Supplement: Supplementary file 1 — Data S1. Supporting Information [file EJN-55-49-s001.docx]

**Supplementary Materials and Methods**

**Apparatus**

The experimental setup consisted of a table supporting a computer monitor which projected visual stimuli on a mirror positioned horizontally in front of participants (see Figure 1a). The monitor (ASUS MG248 24-inch monitor; resolution: 1920 x 1080; refresh rate: 144 Hz) was mounted face down 26.5 cm above the horizontal mirror and the mirror was mounted 26.5 cm above the table. Thus, the visual stimuli appeared to be projected directly onto the surface of the table on the same plane as the hand. A digitizing tablet (GTCO [CAC1] CalComp DB6 1218, Scottsdale, AZ, USA) recorded the position of a hand-held stylus in real-time, which was presented as a cursor on the monitor (white circle; 0.45 cm in diameter) Participants were instructed to control the cursor by sliding the stylus across the tablet with the right hand. The mirror prevented participants from seeing their hands. The cursor position data were acquired at 100 Hz.

Three visual targets were used in each learning session. The target to be reached consisted of a small inner circle surrounded by a grey outer annulus (Figure 1B). The outer annulus had a radius of 2.58 cm, while that of the target was 0.63 cm. For all sessions, the three targets were located along a circular array of 10 cm radius. As a result, the required movement trajectories were 10 cm in length. At the center of the workspace, a grey circle with a radius of 0.42 cm served as the starting point for every trial. It was located at ~35 cm in front of the participants’ chest along the midline.

**Procedures**

To initiate a trial, participants had to bring the cursor into the starting point and remain stationary within its boundary for 250 ms. This prompted the appearance of a target, followed by an auditory Go cue “beep” tone (44.1 kHz), which was presented through headphones (every sound mentioned in the sections below was presented through headphones). The Go cue had an equal pseudo-randomized probability of being presented 300 ms, 500 ms, or 700 ms after the target appeared, instructing participants to initiate their reaching movement. The reason for this was to keep participants engaged in the task. The target presentation was pseudo-randomized so that each target was presented once every 3 trials. Participants were asked to produce straight movements with minimal online corrections in a targeted movement time of approximately 300 ms. This ensured that all participants had a similar speed-accuracy trade-off (Fitts, 1954). Movement onset corresponded to the moment when the cursor left the starting point. Movement end corresponded to when the tangential velocity of the cursor dropped below 0.03 cm/s. Once movements were deemed completed, the cursor’s trajectory during movement as well as its endpoint location were displayed on the screen for 1 200 ms. When participants were provided with vision of the cursor, an auditory cue was always presented at movement end, whereas neither was provided during the NoVision phase (see below). To limit exposure to the visual deviation of the cursor, during the return to the starting point, the cursor was only provided when it was within a 1.39 cm radius around the starting point. On average, five seconds separated each trial. The total duration of a single session varied between approximately 20 and 25 minutes.

**Manipulation of the overlap between the recruited learning-specific neural networks**

The four conditions described below are presented in Figure 1. The objectives of the following conditions were (1) to determine if competing motor memories are required for anterograde interference to emerge (the B 🡪 A vs A 🡪 A conditions) and (2) to assess the extent to which task-specific neural networks need to overlap to generate anterograde interference (the A’ 🡪 A and the α 🡪 A conditions). In the B 🡪 A and A 🡪 A conditions (see Figure 1a and 1b), participants always executed right-handed reaching movements towards targets located in the upper quadrants. Namely, in the B 🡪 A condition, they adapted to a +21° visual deviation in the first session (B) and then to a -21° visual deviation in the second one (A). In the A 🡪 A condition, participants adapted to the same -21° visual deviation twice, that is in both the first and second sessions.

Two additional conditions were carried out to manipulate the commonality of the neural networks between the two learning sessions. In the A’ 🡪 A condition (see Figure 1c), participants executed right-handed reaching movements towards targets located in the lower quadrants in the first session (A’) and the upper during the second session (A). This condition was designed based on work showing that reaching to targets located in different workspaces segregates the directionally-tuned neuronal populations involved in movement preparation and execution (Cowper-Smith et al., 2010; Eisenberg et al., 2010; Fabbri et al., 2010; Haar et al., 2015; Mahan and Georgopoulos, 2013; Tanaka et al., 2018; Toxopeus et al., 2011). Moreover, converging lines of evidence indicate that motor adaptation in one workspace poorly generalizes to other workspaces (Rezazadeh and Berniker, 2019; Schween et al., 2018), indicating that distinct neuronal populations are recruited when visuomotor adaptation occurs in distinct workspaces. It was expected that reaching targets located in different workspaces but with the same effector (same cortical hemisphere) would allow partial segregation of the learning-recruited neural networks in A’ and A, which would attenuate the emergence of anterograde interference during the second session. In the α 🡪 A condition (see Figure 1d), participants first executed left-handed and then right-handed reaching movements towards targets that were always located in the upper quadrants. Doing so was to maximally segregate the learning-recruited neural networks between the two sessions (Bernier et al., 2012; Chang et al., 2008; Gallivan et al., 2011; Levy, 1969; Serrien et al., 2006; Welniarz et al., 2015; Yttri et al., 2014). Moreover, given that between-limb transfer of motor adaptation is limited when the task remains implicit (Malfait and Ostry, 2004; Werner et al., 2019), as would be induced by the present gradual visuomotor adaptation paradigm (Hamel et al., 2017, 2019, 2021), it was expected that left-handed initial learning would attenuate the generation of anterograde interference during right-handed subsequent learning.

Importantly, condition ordering was fully counterbalanced across participants. The choice of the 2 min inter-session interval was based on previous work (Hamel et al., 2021), which showed that such a short inter-session interval is insufficient to prevent initial learning from interfering with subsequent learning.

**Manipulation of target radius to control for hit rates**

To standardize target hit rates across participants and conditions, target size was adjusted for each participant according to their individual level of endpoint accuracy during the Familiarization phase so that they would all achieve at least 50% target hit rates during the Baseline and Acquisition phases of the experiment. Specifically, during Familiarization, all participants reached towards incentivized 0.58 cm radius targets. On these data were fitted confidence circles that encompassed 50% of movement endpoints using a custom-made MATLAB script (Version R2017a; MathWorks Inc.). The radius of the resulting confidence circle was used as the target size for the ensuing learning sessions. Target size was separately adjusted for the right- and left-hand reaching movements towards the targets located in the upper and lower workspace quadrants. Once determined, the target size remained constant for a single condition. To account for potential carry-over effects between experimental sessions, individual accuracy was reevaluated before the start of each condition.

**Delivery of Performance-Contingent Feedback**

For both experiments, visual and auditory feedback was added to the participant’s final cursor position on 50% of trials to increase participants’ arousal and engagement in the task (see Hamel et al. (2019)). Importantly, these manipulations selectively occurred in both the Baseline and Acquisition (both Ramp and Hold) phases, but not during the Retention (both NoVision and Washout) phase. These manipulations were identical for all groups and all sessions.

Namely, at target onset, while the outer annulus and inner target remained grey for 50% of trials, the inner target color turned green for the other 50% of trials (pseudo-randomized). When the target was green, a movement that resulted in a target hit or miss was simultaneously accompanied by a performance-contingent visual (in the form of “Hit” or “Miss”, respectively) and auditory feedback (either a buzzer [44.1 kHz] or “cha-ching” sound [44.1 kHz], respectively). When the target remained grey, no additional visual feedback was provided and the auditory feedback was not contingent upon performance (a “chak-chak” sound [44.1 kHz] that sought to mimic the “cha-ching” sound without conveying any information).

**Statistical analyses**

As mentioned in the Results section, RM ANOVAs were conducted to separately analyze Hand Direction at PV, RT, Endpoint Accuracy, MT, and Hit Rates data. The factors included in the RM ANOVAs were as follows: 4 Conditions (B 🡪 A, A 🡪 A, A’ 🡪 A, α 🡪 A) * 2 Sessions (First, Second) * 3 Phases (Baseline, Acquisition, Retention). If the data violated the assumption of sphericity (Mauchly’s test; *p* < 0.05), the Greenhouse-Geiser correction was applied. ANOVAs were used over their equivalent non-parametric tests because they can handle multifactorial designs, but also because they are robust to deviations from normality (Blanca et al., 2017). Posthoc pairwise comparisons were conducted to decompose significant two-way interactions and main effects (Sawyer, 2009). If data were abnormally distributed during pairwise comparisons (Shapiro-Wilk test; *p* < 0.05), Wilcoxon’s signed-rank test was used over dependent t-tests. The Benjamini-Hochberg procedure(Benjamini and Hochberg, 1995) was used to correct for multiple comparisons during posthoc pairwise comparisons.

**Supplementary Results**

**Condition-specific reach biases evident during the Familiarization phase**

To determine if reach direction differed between the reaching conditions (right arm to upper targets, right arm to lower targets, left arm to upper targets) during Familiarization, the average of Hand Direction at PV (°) of each reaching condition was drawn across all four experimental visits. They were then submitted to a 3 Conditions RM ANOVA, which revealed a main effect of Conditions (F_(2,46)_ = 5.598, *p* = 0.007, $\eta_{p}^{2}$ = 0.196). Pairwise comparisons revealed that the reach direction of the left arm to upper targets (1.01 ± 0.41°) meaningfully differed from the reach direction of both the right arm to upper targets (-0.05 ± 0.40°; t_(23)_ = 2.755, *p* = 0.017, Cohen’s d_z_ = 0.562 [0.126 0.989]) and right arm to lower targets (0.26 ± 0.29°; t_(23)_ = 2.516, *p* = 0.029, Cohen’s d_z_ = 0.514 [0.082 0.935]). The reach direction of the right arm to upper and lower targets did not meaningfully differ (Wilcoxon’s W = 97.0, *p* = 0.136, Cohen’s d_z_ = 0.221 [-0.187 0.624]). Globally, these results suggest that reaching movements executed with the left and right arms were not of similar direction during familiarization, which is likely mediated by the different biomechanical constraints that reaching with different effectors (left vs right arm) imposes (McCrea et al., 2002). Since these differences are likely to confound the subsequent analyses of Hand Direction at PV data during the learning sessions across conditions, these differences were accounted for (see the Methods for details).

**RT and endpoint accuracy systematically improved from the first to the second session**

To determine if the accumulation of fatigue – whether cognitive or physical – covaried with the emergence of anterograde interference, RT, Endpoint Accuracy, MT, and Hit Rates data were examined for signs of performance decrements. Namely, separate 4 Conditions * 2 Sessions * 3 Phases RM ANOVAs were conducted on each kinematic variable. Regarding RT data, the results only revealed a Sessions * Phases interaction (F_(1,23)_= 3.032, *p* = 0.058, $\eta_{p}^{2}$ = 0.117). Pairwise comparisons revealed that RTs were faster in the second Baseline (418 ± 2 ms), Acquisition (415 ± 2 ms), and Retention phases (424 ± 2 ms) as compared to the first Baseline (428 ± 2 ms; t_(23)_ = 4.558, *p* < 0.001, Cohen’s d_z_ = 0.930 [0.442 1.404]), Acquisition (425 ± 2 ms; Wilcoxon’s W = 264.0, *p* < 0.001, Cohen’s d_z_ = 0.912 [0.426 1.383]) and Retention phases (429 ± 2 ms; t_(23)_ = 2.350, *p* = 0.028, Cohen’s d_z_ = 0.480 [0.052 0.898]), respectively. The systematic quickening of RTs in every phase of the second session suggests that fatigue did not accumulate.

Regarding Endpoint Accuracy data, the results revealed a Sessions * Phases interaction (F_(2,46)_ = 5.233, *p* = 0.028, $\eta_{p}^{2}$ = 0.185). Pairwise comparisons revealed that endpoint accuracy systematically improved in the second Baseline (0.50 ± 0.01 cm), Acquisition (0.59 ± 0.01 cm) and Retention phases (1.51 ± 0.03 cm) as compared to the first Baseline (0.57 ± 0.01 cm; t_(23)_ = 5.739, *p* < 0.001, Cohen’s d_z_ = 1.171 [0.641 1.687]), Acquisition (0.66 ± 0.01 cm; t_(23)_ = 8.448, *p* < 0.001, Cohen’s d_z_ = 1.724 [1.080 2.353]) and Retention phases (1.64 ± 0.03 cm; t_(23)_ = 4.297, *p* < 0.001, Cohen’s d_z_ = 0.877 [0.397 1.343]), respectively. This systematic improvement in accuracy at movement endpoint in every phase of the second session suggests that fatigue did not accumulate.

**Movement speed either improved or remained stable in the second session**

Regarding MT data, the results revealed a Conditions * Sessions (F_(3,69)_ = 4.148, *p* = 0.009, $\eta_{p}^{2}$ = 0.153) and Sessions * Phases interactions (F_(2,46)_ = 12.767, *p* < 0.001, $\eta_{p}^{2}$ = 0.357). These interactions are separately broken down below.

Concerning the Conditions * Sessions interaction, pairwise comparisons revealed that MT were slower in the first session (329 ± 4 ms) than in the second one (316 ± 4 ms) in the α 🡪 A condition (t_(23)_ = 2.738, *p* = 0.048, Cohen’s d_z_ = 0.559 [0.123 0.985]). Such a difference between sessions was not found in the B 🡪 A (t_(23)_ = 0.728, *p* = 0.948, Cohen’s d_z_ = 0.149 [-0.255 0.549]), A 🡪 A (t_(23)_ = 0.080, *p* = 0.937, Cohen’s d_z_ = 0.016 [-0.384 0.416]), or A’ 🡪 A (t_(23)_ = 0.189, *p* = 1.000, Cohen’s d_z_ = 0.039 [-0.362 0.438]). Globally, this interaction revealed that participants executed slower movements when learning with their left hand as compared to learning with their right hand. These results also indicate either improvements or stability in movement speed across sessions, which suggests that fatigue did not accumulate upon the second session.

Concerning the Sessions * Phases interaction, pairwise comparisons revealed that MT tended to decrease in the second Acquisition (308 ± 3 ms) as compared to the first one (315 ± 3 ms; Wilcoxon W = 223, *uncorrected* *p* = 0.037, Cohen’s d_z_ = 0.496 [0.067 0.916]). However, this difference did not survive the correction for multiple comparisons (corrected *p* = 0.110). Such a difference between sessions was not found for both the Baseline (t_(23)_ = 1.182, *p* = 0.249, Cohen’s d_z_ = 0.241 [-0.167 0.645] and Retention phases (t_(23)_ = 1.572 , *p* = 0.195, Cohen’s d_z_ = 0.321 [-0.093 0.728]). Globally, the improvements or stability in movement speed execution suggest that fatigue did not accumulate upon the second session.

**Hit Rates either improved or remained stable in the second session**

Regarding Hit Rates, the results revealed a Sessions * Phases interaction (F_(2,46)_ = 13.301, *p* < 0.001, $\eta_{p}^{2}$ = 0.366). Pairwise comparisons revealed that Hit Rates increased during the second Baseline (71.2 ± 1.2%) and Acquisition (60.7 ± 0.9%) as compared to the first Baseline (66.5 ± 1.2%; t_(23)_ = 3.929, *p* = 0.001, Cohen’s d_z_ = 0.802 [0.334 1.257]) and Acquisition phases (55.2 ± 0.9%; t_(23)_ = 6.289, *p* < 0.001, Cohen’s d_z_ = 1.284 [0.732 1.820]), respectively. Hit Rates did not meaningfully increase from the first Retention phase (29.5 ± 0.8%) to the second one (30.3 ± 0.8 %; t_(23)_ = 1.032, *p* = 0.313, Cohen’s d_z_ = 0.211 [-0.196 0.613]). Globally, these results indicate that Hit Rates either improved or remained stable across sessions, which suggests that fatigue did not accumulate upon the second session.

**Separating the Acquisition phase into its constitutive Ramp and Hold phases revealed similar results**

To determine if participants differed in their adaptation levels across sessions during the Ramp and Hold phases, an additional 4 Conditions * 2 Sessions * 2 Phases (Ramp, Hold) RM ANOVA was conducted. The results revealed no Conditions * Sessions * Phases interaction (F_(3,69)_ = 0.216, *p* = 0.885, $\eta_{p}^{2}$ = 0.009), but revealed a Conditions * Sessions interaction (F_(3,69)_ = 2.664, *p* = 0.0546, $\eta_{p}^{2}$ = 0.104). Pairwise comparisons revealed that adaptation levels did not differ across sessions for the A 🡪 A (t_(23)_ **=** 0.273, *p* = 0.7874, Cohen’s d_z_ = 0.056 [-0.345 0.456]), A’ 🡪 A (t_(23)_ **=** 1.280, *p* = 0.2844, Cohen’s d_z_ = 0.261 [-0.149 0.666]), and the α 🡪 A conditions (t_(23)_ = 1.289, *p* = 0.4204, Cohen’s d_z_ = 0.263, [-0.147 0.668]). However, adaptation levels were lower in the second sessions as compared to the first one in the B 🡪 A condition (t_(23)_ = 2.746, *p* = 0.0460, Cohen’s d_z_ = 0.561 [0.124 0.987]). Globally, these results indicate that adaptation levels during both the Ramp and Hold phases are lower in the second session as compared to the first one in the B 🡪 A condition only. These results are quantitatively similar to the analyses conducted on Acquisition (as an average measure of both Ramp and Hold) reported in the manuscript.

**Separating the Retention phase into its constitutive NoVision and Washout phases revealed similar results**

To determine if participants differed in their aftereffect levels across sessions during the NoVision and Washout phases, an additional 4 Conditions * 2 Sessions * 2 Phases (NoVision, Washout) RM ANOVA was conducted. The results revealed no Conditions * Sessions * Phases interaction (F_(3,69)_ = 1.723, *p* = 0.170, $\eta_{p}^{2}$ = 0.070), but revealed a Conditions * Sessions interaction (F_(3,69)_ = 5.054, *p* = 0.0032, $\eta_{p}^{2}$ = 0.180). Pairwise comparisons revealed that the aftereffect levels were lower in the second session as compared to the first one in both the B 🡪 A (W = 300, *p* < 0.001, Cohen’s d_z_ = 1.385 [0.813 1.942]) and A 🡪 A conditions (t_(23)_ = 3.374, *p* = 0.005, Cohen’s d_z_ = 0.689 [0.236 1.129]). The aftereffect levels were marginally lower in the second session as compared to the first one in the A’ 🡪 A condition (t_(23)_ = 2.018, *p* = 0.0739, Cohen’s d_z_ = 0.412 [-0.009 0.825]), but did not differ in the α 🡪 A condition (t_(23)_ = 1.335, *p* = 0.195, Cohen’s d_z_ = 0.272 [-0.138 0.677]).

**References**

Benjamini, Y., Hochberg, Y., 1995. Controlling the False Discovery Rate: A Practical and Powerful Approach to Multiple Testing. J. R. Stat. Soc. Ser. B Methodol. 57, 289–300.

Bernier, P.-M., Cieslak, M., Grafton, S.T., 2012. Effector selection precedes reach planning in the dorsal parietofrontal cortex. J. Neurophysiol. 108, 57–68. https://doi.org/10.1152/jn.00011.2012

Blanca, M.J., Alarcón, R., Arnau, J., Bono, R., Bendayan, R., 2017. Non-normal data: Is ANOVA still a valid option? Psicothema 29, 552–557. https://doi.org/10.7334/psicothema2016.383

Chang, S.W.C., Dickinson, A.R., Snyder, L.H., 2008. Limb-Specific Representation for Reaching in the Posterior Parietal Cortex. J. Neurosci. 28, 6128–6140. https://doi.org/10.1523/JNEUROSCI.1442-08.2008

Cowper-Smith, C.D., Lau, E.Y.Y., Helmick, C.A., Eskes, G.A., Westwood, D.A., 2010. Neural coding of movement direction in the healthy human brain. PloS One 5, e13330. https://doi.org/10.1371/journal.pone.0013330

Eisenberg, M., Shmuelof, L., Vaadia, E., Zohary, E., 2010. Functional Organization of Human Motor Cortex: Directional Selectivity for Movement. J. Neurosci. 30, 8897–8905. https://doi.org/10.1523/JNEUROSCI.0007-10.2010

Fabbri, S., Caramazza, A., Lingnau, A., 2010. Tuning Curves for Movement Direction in the Human Visuomotor System. J. Neurosci. 30, 13488–13498. https://doi.org/10.1523/JNEUROSCI.2571-10.2010

Fitts, P.M., 1954. The information capacity of the human motor system in controlling the amplitude of movement. J. Exp. Psychol. 47, 381–391.

Gallivan, J.P., McLean, D.A., Smith, F.W., Culham, J.C., 2011. Decoding Effector-Dependent and Effector-Independent Movement Intentions from Human Parieto-Frontal Brain Activity. J. Neurosci. 31, 17149–17168. https://doi.org/10.1523/JNEUROSCI.1058-11.2011

Haar, S., Donchin, O., Dinstein, I., 2015. Dissociating Visual and Motor Directional Selectivity Using Visuomotor Adaptation. J. Neurosci. 35, 6813–6821. https://doi.org/10.1523/JNEUROSCI.0182-15.2015

Hamel, R., Côté, K., Matte, A., Lepage, J.-F., Bernier, P.-M., 2019. Rewards interact with repetition-dependent learning to enhance long-term retention of motor memories. Ann. N. Y. Acad. Sci. 1452, 34–51. https://doi.org/10.1111/nyas.14171

Hamel, R., Dallaire-Jean, L., De La Fontaine, É., Lepage, J.F., Bernier, P.M., 2021. Learning the same motor task twice impairs its retention in a time- and dose-dependent manner. Proc. R. Soc. B Biol. Sci. 288, 20202556. https://doi.org/10.1098/rspb.2020.2556

Hamel, R., Trempe, M., Bernier, P.-M., 2017. Disruption of M1 activity during performance plateau impairs consolidation of motor memories. J. Neurosci. Off. J. Soc. Neurosci. https://doi.org/10.1523/JNEUROSCI.3916-16.2017

Levy, J., 1969. Possible Basis for the Evolution of Lateral Specialization of the Human Brain. Nature 224, 614–615. https://doi.org/10.1038/224614a0

Mahan, M.Y., Georgopoulos, A.P., 2013. Motor directional tuning across brain areas: directional resonance and the role of inhibition for directional accuracy. Front. Neural Circuits 7. https://doi.org/10.3389/fncir.2013.00092

Malfait, N., Ostry, D.J., 2004. Is interlimb transfer of force-field adaptation a cognitive response to the sudden introduction of load? J. Neurosci. Off. J. Soc. Neurosci. 24, 8084–8089. https://doi.org/10.1523/JNEUROSCI.1742-04.2004

McCrea, P.H., Eng, J.J., Hodgson, A.J., 2002. Biomechanics of reaching: clinical implications for individuals with acquired brain injury. Disabil. Rehabil. 24, 534–541. https://doi.org/10.1080/09638280110115393

Rezazadeh, A., Berniker, M., 2019. Force field generalization and the internal representation of motor learning. PLOS ONE 14, e0225002. https://doi.org/10.1371/journal.pone.0225002

Sawyer, S.F., 2009. Analysis of Variance: The Fundamental Concepts. J. Man. Manip. Ther. 17, 27E-38E. https://doi.org/10.1179/jmt.2009.17.2.27E

Schween, R., Taylor, J.A., Hegele, M., 2018. Plan-based generalization shapes local implicit adaptation to opposing visuomotor transformations. J. Neurophysiol. 120, 2775–2787. https://doi.org/10.1152/jn.00451.2018

Serrien, D.J., Ivry, R.B., Swinnen, S.P., 2006. Dynamics of hemispheric specialization and integration in the context of motor control. Nat. Rev. Neurosci. 7, 160–166. https://doi.org/10.1038/nrn1849

Tanaka, H., Miyakoshi, M., Makeig, S., 2018. Dynamics of directional tuning and reference frames in humans: A high-density EEG study. Sci. Rep. 8, 8205. https://doi.org/10.1038/s41598-018-26609-9

Toxopeus, C.M., de Jong, B.M., Valsan, G., Conway, B.A., Leenders, K.L., Maurits, N.M., 2011. Direction of movement is encoded in the human primary motor cortex. PloS One 6, e27838. https://doi.org/10.1371/journal.pone.0027838

Welniarz, Q., Dusart, I., Gallea, C., Roze, E., 2015. One hand clapping: lateralization of motor control. Front. Neuroanat. 9. https://doi.org/10.3389/fnana.2015.00075

Werner, S., Strüder, H.K., Donchin, O., 2019. Intermanual transfer of visuomotor adaptation is related to awareness. PLoS ONE 14. https://doi.org/10.1371/journal.pone.0220748

Yttri, E.A., Wang, C., Liu, Y., Snyder, L.H., 2014. The parietal reach region is limb specific and not involved in eye-hand coordination. J. Neurophysiol. 111, 520–532. https://doi.org/10.1152/jn.00058.2013
